# Crosstalk between melatonin and reactive oxygen species in fruits and vegetables post-harvest preservation: An update

**DOI:** 10.3389/fnut.2023.1143511

**Published:** 2023-03-03

**Authors:** Na Li, Kefeng Zhai, Qin Yin, Quan Gu, Xingtao Zhang, Merced G. Melencion, Ziping Chen

**Affiliations:** ^1^Biology Department, Center for Biodiversity Research and Extension in Mindanao, Central Mindanao University, Musuan, Philippines; ^2^School of Biological and Food Engineering, Suzhou University, Suzhou, China; ^3^Engineering Research Center for Development and High Value Utilization of Genuine Medicinal Materials in North Anhui Province, Suzhou, China; ^4^School of Biology, Food and Environment, Hefei University, Hefei, China; ^5^Anhui Promotion Center for Technology Achievements Transfer, Anhui Academy of Science and Technology, Hefei, China

**Keywords:** fruit, melatonin, post-harvest preservation, reactive oxygen species, signaling networks, vegetable

## Abstract

Fruits and vegetables contain numerous nutrients, such as vitamins, minerals, phenolic compounds, and dietary fibers. They reduce the incidence of cardiovascular diseases and the risk of certain chronic diseases, and improve the antioxidant and anti-inflammatory capacity. Moreover, melatonin was found in various fruits and vegetables species. Melatonin acts as a multifunctional compound to participate in various physiological processes. In recent years, many advances have been found that melatonin is also appraised as a key modulator on the fruits and vegetables post-harvest preservation. Fruits and vegetables post-harvest usually elicit reactive oxygen species (ROS) generation and accumulation. Excess ROS stimulate cell damage, protein structure destruction, and tissue aging, and thereby reducing their quality. Numerous studies find that exogenous application of melatonin modulates ROS homeostasis by regulating the antioxidant enzymes and non-enzymatic antioxidants systems. Further evidences reveal that melatonin often interacts with hormones and other signaling molecules, such as ROS, nitric oxide (NO), hydrogen sulfide (H_2_S), and etc. Among these ‘new’ molecules, crosstalks of melatonin and ROS, especially the H_2_O_2_ produced by RBOHs, are provided in fruits and vegetables post-harvest preservation in this review. It will provide reference for complicated integration of both melatonin and ROS as signal molecules in future study.

## Introduction

Fruits and vegetables contain numerous nutrients, such as vitamins, minerals, phenolic compounds, and dietary fibers (1–4). They play an essential part of a well-balanced daily food. It is generally recommended to eat more fruits and vegetables to reduce the incidence of cardiovascular diseases and the risk of certain chronic diseases, and improve the antioxidant and anti-inflammatory capacity (3, 5). For example, polyphenols inhibit chronic inflammation through regulating multiple inflammation-associated cell signaling pathways (6). However, fruits and vegetables often generate significant post-harvest losses after harvest (3). They are vulnerable to mechanical damages, water and phytochemicals loss, microbial infections, thus resulting in a considerable concern during long-term storage (7, 8). To reduce post-harvest losses, several appropriate storage technologies are used, including cold chain management, hypobaric storage, modified atmosphere package (MAP), and ultraviolet treatment (9–13). To some extent, natural/synthetic preservative agent can also preserve fruits and vegetables storage, whereas there are some residues of chemical compounds (14). To date, previous studies also indicate that plant natural hormones (melatonin, ethylene (ET), salicylic acid (SA), and methyl jasmonate (MeJA), etc) and signaling molecules (nitric oxide (NO), hydrogen sulfide (H_2_S), and reactive oxygen species (ROS), etc) can play key roles in regulating the maturation and senescence of fruits and vegetables, delaying postharvest senescence and extending shelf life (15–21).

Acting as a pleiotropic compound, melatonin (*N*-acetyl-5-methoxytryptamine) has a wide range of cellular and physiological functions in living organisms (22–24). For example, melatonin modulates sleep and circadian rhythms, enhances immunity and anti-inflammatory activities (23, 24). Melatonin improves the anti-inflammatory activity, particularly against the chronic inflammation which induced by many chronic diseases (25). In plants, melatonin was firstly detected in 1995 (26, 27). Since then, it was found in various plant species and their different tissue parts, such as rice, wheat, tomato, apple, strawberry, grape, pepper, cucumber, and solanaceous, etc (28–36). Melatonin acts a key molecule to mediate multiple physiological processes, such as the alleviation of abiotic and biotic stresses, and plant growth and development (37–42). For example, melatonin obviously promoted the lateral root formation in *Arabidopsis thaliana* (37). Recently, many studies have reported that melatonin plays an vital role in the fruit and vegetable post-harvest preservation (43–46). In general, endogenous melatonin was increased by exogenous application of melatonin in broccoli, pear, and *Zizyphus* jujuba fruit (43, 44, 46). Then, melatonin observably decreased the accumulation of ROS by enhancing antioxidant capacity and total phenolic and ascorbic acid (AsA) content, and improved the quality of fruits and vegetables (43, 44, 46). Besides, melatonin improved the polyphenol accumulation and antioxidant capacity *via* ethylene signaling in grape berries (47).

ROS contain a group of molecules, mainly including hydrogen peroxide (H_2_O_2_), hydroxyl radical (OH), superoxide anion (O_2_^•–^), and singlet oxygen (^1^O_2_) (48). ROS can cause the oxidation of lipids, and damages of proteins and many other small molecules structures (48). Accordingly, plants have evolved sophisticated antioxidant strategies to regulate the ROS homeostasis, such as antioxidant enzymes [catalase (CAT), ascorbate peroxidase (APX), superoxide dismutase (SOD), and glutathione peroxidase (GPX)] and non-enzymatic antioxidants (glutathione (GSH), AsA, flavonoids, carotenoids, and alkaloids, etc) (40, 41). Moreover, numerous studies revealed that ROS play key dual roles in the signaling networks in plant stress responses and developmental processes (49, 50). Interestingly, several studies have revealed that the signaling crosstalk between melatonin and ROS was also suggested in red pear and strawberry fruits during post-harvest period (51, 52).

In this review, we mainly discuss exogenous application of melatonin in fruits and vegetable preservation, synthesis of endogenous melatonin, effects of melatonin on the quality of postharvest fruits and vegetable, and the mechanism of melatonin-modulated postharvest protection of fruits and vegetables. We further highlight and discuss the vital role of ROS signaling during the processes, so as to provide reference for future complicated integration of both melatonin and ROS as signal molecules.

## The changes of phenomenon and quality of fruits and vegetables during the postharvest period

Fruits and vegetables contain diverse nutrients, such as phenolic compounds, AsA, carotenoids, and mineral content, which beneficial for the anti-nflammation, antioxidation, anti-diabetes, cancer prevention, and cardio-protection in human (1, 2). Many popular kinds of fruits and vegetables, such as tomato, apple, banana, papaya, etc., are consumed worldwide with the rapidly increasing demand and production. However, most of these are highly susceptible to soften rapidly and over-ripen, and often accompanying by the chlorophyll degradation and pathogens (53–59). For example, papaya ripened and softened rapidly, and the fruit peel color gradually turned from green to yellow after harvest (53). Meanwhile, the lightness value declined slightly, the chroma value increased, and the hue angle value gradually dropped during late storage. The most serious damage was disease incidence, and thus decreasing the papaya commodity rate. Similar changes of fruit firmness, hue angle, brightness, and color saturation values were also found in guava during the postharvest period (54). After harvest for 11 days, the anthracnose disease index and disease incidence increased rapidly. In cherry tomato and litchi fruits, the weight loss and fruit firmness were declined, accompanied by fruit decay during storage (57, 59). Furthermore, other fruits and vegetables usually encountered the same cases as well (56, 57, 60). Hence, low-temperature preservation for fruits and vegetables has received increasing research attention (61). Nevertheless, storage for long times may cause chilling injury, such as surface pitting and browning, inability to ripen, watersoaking lesions, and rapid decay (62, 63).

## The changes of melatonin content in fruits and vegetables during the postharvest period

Our previous reviews systematically summarized the melatonin biosynthesis and catabolism in plant tolerance to abiotic stresses (38, 40–42). In general, various abiotic stresses, such as salinity, heat, cold, drought, and cadmium metal stresses induce melatonin accumulation by the upregulation of genes which encoding tryptamine 5-hydroxylase (T5H), tryptophan decarboxylase (TDC), *N*-acetylserotonin methyltransferase (ASMT), serotonin *N*-acetyltransferase (SNAT), and caffeic acid O-methyltransferase (COMT) (40). Interestingly, the changes of melatonin content have different trends among different kinds of fruits and vegetables, and some findings were listed in Table 1 and (19, 44, 58, 64–71, 73). Wang et al. (19) found that endogenous melatonin was increased at 0 d to 14 d, and decreased at 14 d to 63 d throughout storage period in cherry fruit. Interestingly, it was decreased dramatically from anthesis to maturity period (45). These results suggested that endogenous melatonin accumulation was regulated by growing and picking storage periods in fruits. Similarly, melatonin content of table grape, mango, cassava, and strawberry was in parallel with the change trend of cherry fruit, and manifested a trend of rising first and then falling (64–66, 69). Nevertheless, in “Summer black” grape, the change of melatonin accumulation showed an contrary tendency (67). Besides, it showed an decreasing trend in angeleno plum, pakchoi, and cherry tomato (70, 71, 73). Moreover, expression of the genes *TDCs*, *T5Hs*, *SNATs*, and *ASMTs* related to melatonin biosynthesis were also differently regulated in table grape, mulberry fruits, cassava, strawberry, and cherry tomato (64–66, 68, 73). Therefore, melatonin accumulation and its biosynthesis genes transcripts are dynamic and highly regulated in various fruits and vegetables during the post-harvest period.

**Table 1:** Summary table explaining the changes of melatonin content, and genes related to melatonin metabolic pathway in fruits and vegetables during the postharvest period.

Fruit Species	Impact on melatonin content, or/and genes and enzyme activities related to melatonin metabolic pathway	References
Cassava	Melatonin (0–2 h ↑; 2–72 ↓); *TCD1*, *TCD2*, *T5H*, *ASMT1*, *ASMT2*, *ASMT3*, *SNAT*	(64)
Strawberry	Melatonin (0–3 d ↑; 3–12 ↓); *TCD*, *T5H*, *ASMT*, *SNAT*	(65)
Sweet cherry	Melatonin (0–14 d ↑; 14–63 d↓)	(19)
Jujube	Melatonin (0, 14, 28 d no significant changes)	(44)
“Feizixiao” litchi	Melatonin (0–12 d ↑)	(58)
Table grape	Melatonin (0–15 d ↑; 15–25 d↓), 5-methoxytryptamine (5-MT) (0–15 d ↑; 15–25 d↓); *TDC1*, *TDC2*, *TDC3*, *TDC4*, *T5H1*, *T5H2*, *T5H3*, *T5H4*, *T5H5*, *SNAT1*, *SNAT2*, *SNAT3*, *ASMT1*, *ASMT2*, *ASMT3*, *ASMT4*	(66)
“Summer black” grape	Melatonin (0–40 d ↓; 40–50 d ↑)	(67)
Mulberry	*ASMT4*, *ASMT20* genes	(68)
Mango	Melatonin (0–14 d ↑; 14–28 d↓)	(69)
Angeleno plum	Melatonin (0–8 d↓)	(70)
Pakchoi	Melatonin (0–8 d↓)	(71)
Cherry tomato	Melatonin (0–72 h↓); T*CD*, *T5H*, *ASMT*, *SNAT*	(72)

TDC, tryptophan decarboxylase; T5H, tryptamine 5-hydroxylase; SNAT, serotonin N-acetyltransferase; ASMT, N-acetylserotonin methyltransferase.

## Protective effects of exogenous melatonin on qualities of fruits and vegetables during the postharvest period

Previous studies have shown that hormones, such as ET, SA, gibberellins [GAs, including gibberellin 1 (GA1), gibberellin 3 (GA3), gibberellin 4 (GA4), and gibberellin 7 (GA7)], MeJA, and abscisic acid (ABA), modulate the postharvest preservation of fruits and vegetables (70, 74–77). Over the past several years, numerous reports have proposed that melatonin acts as an important role on qualities of fruits and vegetables during the postharvest period (53, 54, 56–60, 67, 76). For example, exogenous melatonin treatments delayed fruit firmness decrease, maintained higher hue of the peel fruit, and retained greater lightness of papayas than the control group during the later storage period (53, 54). Similarly, it observably alleviated the decrease of fifirmness and the weight loss in cherry tomato (59). Fruit colour index (a*/b*) was also obviously increased by melatonin treatment in both sweet cherry and guava fruits (78). In pepper, broccoli, and Chinese flowering cabbage vegetables, exogenous melatonin application inhibited the degradation of chlorophyll during the postharvest period (43, 56, 79). In addition to the above phenotypic changes, melatonin also reduced the decay and disease index in fruits (41, 53, 54, 80). Moreover, exogenous melatonin also bought about significant increases in total soluble solids, sugar, protein, AsA, carotenoids, and total flavonoid and phenols contents, which were important substances of fruits and vegetables (43, 56, 81–83). Besides, melatonin mediated the aroma volatiles (propyl acetate and hexyl acetate) of postharvest pear fruit (84, 85).

## Effects of exogenous melatonin on the redox homeostasis of fruits and vegetables during the postharvest period

In general, ROS (mainly MDA, H_2_O_2_, and O_2_^•–^) are largely caused during fruit ripening period, and induce oxidizing proteins and membrane lipids formation (53). For example, O_2_^•–^ produce by the oxygen reduction by the electron transport chain (ETC) (53, 54). They also generate by photorespiration pathway and fatty acid-oxidation reaction (59). Then, H_2_O_2_ produces from O_2_^•–^ by the activity of SOD and/or glycolate oxidases. Moreover, NADPH oxidases, polyamine oxidases (PAO), and cell wall bound peroxidases (POX) induce the ROS generation in cell membrane, cell wall, and apoplast, respectively (7, 57, 58). As toxic byproducts, ROS could cause serious damages to proteins and quality of fruits and vegetables. Combined with the antioxidant capacity of melatonin, these led to study the role of melatonin in the postharvest preservation of fruits and vegetables, especially in recent years (86–110). In this review, the protective impacts of melatonin on the antioxidant capacity of fruits and vegetables during the postharvest period have been summarized in Table 2. In fact, ROS were largely stimulated in fruits and vegetables, including papaya, cherry tomato, pepper, wax apple, Chinese flowering cabbage, pear, peach, litch, pomegranate, sweet cherry, sapota, apple, blueberry, longan, zucchini, guava, rambutan, water bamboo shoot, mango, tomato, eggplant, rosa roxburghii fruit, cucumber, jujube, sweetpotato, avocado, persimmons, and table grape during the postharvest period (stored at room temperature and/or low temperature; Table 2). Then, the ROS accumulation were significantly decreased by exogenous application of melatonin. Two main pathways might be involved in melatonin-inhibited ROS acumulation. Exogenous application of melatonin improved the antioxidant contents, such as GSH, AsA, proline, flavonoids, carotenoids, anthocyanins, and dehydroascorbate (DHA) through inducing the expression of *GSH*, *GR1*, *GR2*, *GMDH*, *GME*, *GGGT*, *GPP*, *GDH*, and *GLDH* genes (Table 2) and (88, 92, 95, 101). In most of the above fruits and vegetables, the antioxidant enzymes act as key roles in melatonin-downregulated ROS overproduction, such as CAT, SOD, APX, GR, GPX, DHAR, and MDHAR (Table 2). Besides, exogenous application of melatonin enhanced the total antioxidant capacity (T-AOC), cupric-reducing antioxidant power (CUPRAC), ferric-reducing antioxidant power (FRAP), trolox equivalent antioxidant capacity (TEAC), ferric reducing antioxidant power (FRAP), and 1,1-diphenyl-2-trinitrophenylhydrazine (DPPH) and 2,2′-azino-bis (3-ethylbenzothiazoline-6-sulfonic acid) (ABTS) radical scavenging capacity. For example, exogenously melatonin obviously induced the expression of *PpAPXs*, *PpSODs*, and *PpCATs*, and thereby activating the antioxidant system in peach fruit during storage for 14 d (88). Furthermore, the expression of AsA biosynthetic genes (including *GMDH*, *GME*, *GGGT*, *GPP*, *GDH*, and *GLDH*) were also stimulated, which increase the content of AsA to inhibit the ROS accumulation (88). In addition, exogenous melatonin interacted with ROS by regulating the expression of genes involved in AsA-GSH cycle, such as *DHA*, *DHAR*, *MDHAR*, *GSH*, *GSSG*, and *GR* in sweet cherry (101). Among the above fruits, blueberry contains high level of bioactive compounds, flavonoids and anthocyanins. These were also increased by exogenous melatonin to improve the nutraceutical traits of blueberry fruit during storage time (98).

**Table 2:** Summary table explaining the impacts of exogenous melatonin on the antioxidative defense systems of fruits and vegetables during the postharvest period.

Fruit and vegetable names	Treatments	Impact on oxidative markers and antioxidative defense systems	References
Papaya	0, 100, 400, and 800 μM melatonin	H_2_O_2_, MDA, O_2_^·−^; SOD, CAT, POD, APX, GR, NOX, T-AOC, AsA, flavonoids	(53)
Cherry tomato	0 and 100 μM melatonin	MDA; GSH, AsA, GPX, APX, GR, T-AOC	(59)
Pepper	0 and 100 μM melatonin	H_2_O_2_, MDA, O_2_^·−^; AsA, DHA, GSH, GSSG, APX, SOD, CAT, POD, GR, MDHAR, DHAR	(56)
Wax apple	0, 800 μM melatonin	MDA, H_2_O_2_, O_2_^·−^; SOD, CAT, APX, GR; *CAT1*, *SOD2*	(60)
Chinese flowering cabbage	0 and 100 μM melatonin	H_2_O_2_, MDA, O_2_^·−^; POD, SOD, CAT, APX, GR, DHAR, MDHAR, AsA, DHA, GSH, GSSG; *RBOHB*, *RBOHC*, *RBOHD*, *RBOHD2*, *RBOHE*, *POD*, *SOD*, *CAT*, *APX*, *GR*, *DHAR*, *MDHAR*	(79)
Pear	0, 50, 100, 150, 200, and/or 500 μM melatonin	H_2_O_2_, MDA; SOD, POD, AsA, DPPH and ABTS scavenging capacity; *POD*	(46, 85)
Peach	0 and 100 μM melatonin	MDA, H_2_O_2_, O_2_^·−^; AsA, GSH, POD; *SOD1*, *SOD2*, *SOD3*, *SOD4*, *SOD5*, *SOD6*, *SOD7*, *SOD8*, *CAT1*, *CAT2*, *APX1*, *APX3*, *APX6*, *MDHAR1*, *MDHAR2*, *DHAR2*, *DHAR3*, *GR1*, *GR2*, *GMDH*, *GME*, *GGGT*, *GPP*, *GDH*, *GLDH*	(88, 92)
Litch	0, 50, 100, 200, and/or 600 μM melatonin	MDA, H_2_O_2_, O_2_^·−^; flavonoids, anthocyanin, proline, P5CS, PDH, POD, SOD, CAT, APX, GR; *Fe-SOD*	(58, 95)
Pomegranate	0 and 100 μM melatonin	AsA, AOX, AAO, APX, GR, GSH, anthocyanins	(86)
Sweet cherry	0, 50, 100, 150, 200, 300, and/or 500 μM melatonin	MDA, H_2_O_2_, O_2_^·−^; SOD, CAT, APX, POD, DHAR, GR, MDHAR, AsA, DHA, GSH, GSSG, flavonoids, anthocyanins; *Cu/Zn-SOD*, *Mn-SOD*, *CAT*, *APX*, *MDHA*, *MDHAR*, *DHA*, *DHAR*, *GSH*, *GSSG*, *GR*	(19, 45, 78, 101)
Sapota	0, 30, 60, and 90 μM melatonin	MDA, O_2_^·−^, H_2_O_2_; proline, SOD, CAT	(102)
Apple	0 and 1 mM melatonin	MDA; CAT, SOD, POD	(104)
Blueberry	0 and 1 mM melatonin	H_2_O_2_, MDA; polyphenols, flavonoids, anthocyanins, AsA, SOD, CAT, APX, POD	(98)
Longan	0 and 400 μM melatonin	H_2_O_2_, MDA, O_2_^·−^; POD, PPO, flavonoids, SOD, CAT, APX, AsA, GSH	(97)
Zucchini	0 and 1 mM melatonin	MDA	(100)
Guava	0, 50, 100, 150, 200, 400, and/or 600 μM melatonin	H_2_O_2_, MDA, O_2_^·−^; SOD, APX, CAT, T-AOC, AsA, flavonoids, total soluble sugar	(54, 89)
Rambutan	0 and 125 μM melatonin	H_2_O_2_, MDA, O_2_^·−^; AsA, DHA, GSH, GSSG, POD, PPO, SOD, CAT, flavonoids, anthocyanins, APX, GR, MDHAR, DHAR	(107)
Water bamboo shoot	0 and 500 μM melatonin	AsA, POD; *POD1*, *POD2*, *POD3*, *POD4*, *POD5*	(108)
Mango	0, 100, or 200 μM melatonin	H_2_O_2_, MDA, O_2_^·−^; carotenoid, SOD, CAT, POD, APX, CUPRAC, TEAC, DPPH, TEAC, FRAP	(69, 87, 91)
Tomato	0 and 10 μM melatonin	SOD, CAT, POD, APX, GSH	(109)
Eggplant	0, 50, 100, 150, and 200 μM melatonin	H_2_O_2_, MDA; SOD, CAT, anthocyanins; *SOD*, *CAT1*, *CAT2*	(111)
Rosa roxburghii fruit	0, 20, 50, 100, 200, and 400 μM melatonin	H_2_O_2_; SOD, CAT, POD, APX, GR, MDHAR, DHAR, AsA, GSH; *APX*, *GR*, *MDHAR*, *DHAR*	(90)
Cucumber	0, 50, 100, and 500 μM melatonin	H_2_O_2_, MDA, O_2_^·−^; AsA, proline	(96)
Jujube	0, 20, 50, 100, 200, and 400 μM melatonin	H_2_O_2_, MDA, O_2_^·−^; AsA, GSH, APX, SOD, CAT, POD,	(106, 110)
Sweetpotato	0, 200, and 500 μM melatonin	H_2_O_2_, MDA, O_2_^·−^; SOD, CAT, POD, APX, GR, AsA, vitamin C, *SOD1*, *SOD2*, *CAT1*, *APX1*, *APX3*, *GR1*, *GR2*, *DHAR*	(94)
Avocado	0 and 1 mM melatonin	H_2_O_2_, MDA, O_2_^·−^; SOD, CAT, APX, POD, flavonoids, AsA	(99)
Persimmons	0 and 100 μM melatonin	H_2_O_2_, MDA; flavonoids, AsA, DPPH and ABTS radical scavenging activity, FRAP	(93)
Table grape	0, 50, and 100 μM melatonin	H_2_O_2_, O_2_^·−^; CAT, POD	(103)

H_2_O_2_, hydrogen peroxide; MDA, malondialdehyde; O_2_^•–,^ superoxide anion; Cu/Zn-SOD, copper/zinc-superoxide dismutase; Mn-SOD, manganese-superoxide dismutase; POD, guaiacol peroxidase; APX, ascorbate peroxidase; GR, glutathione reductase; T-AOC, total antioxidant capacity; AsA, ascorbic acid; GSH, reduced glutathione; GPX, glutathione peroxidase; DHA, dehydroascorbate; GSSG, oxidized glutathione; CAT, catalase; MDHA; monodehydroascorbate reductase; MDHAR, monodehydroascorbate; DHAR, dehydroascorbate reductase; RBOH, respiratory burst oxidase homologue; DPPH, 1,1-diphenyl-2-trinitrophenylhydrazine; ABTS, 2,2′-azino-bis (3-ethylbenzothiazoline-6-sulfonic acid); NOX, NADH oxidase; GMPH, mannose-1-phosphate guanylyltransferase; GME, GDP-D-mannose-3′,5′-epimerase; GGGT, GDP-L-galactose guanylyltransferase; GPP, L-galactose-1-phosphate phosphatase; GDH, L-galactose-1-dehydrogenase; GLDH, L-galactono-1,4-lactone dehydrogenase; P5CS, Δ1-pyrroline-5-carboxylate synthetase; PDH, pyruvate dehydrogenase; CUPRAC, Cupric-reducing antioxidant power; FRAP, Ferric-reducing antioxidant power; TEAC, Trolox equivalent antioxidant capacity; FRA, ferric reducing antioxidant power.

## The roles of hormones in melatonin-modulated postharvest protection of fruits and vegetables during storage period

In recent years, hormones have been described to regulate fruits and vegetables postharvest performance (112). For example, ET and ABA played central roles in modulating senescence that strongly influence fruits and vegetables shelf-life (21, 113, 114). Ethylene is synthesized from *S*-adenosylmethionine to 1-aminocyclopropane-1-carboxylate (ACC) by ACC synthase (ACS), and then ACC is oxidazed by ACC oxidase (ACO) (21). Thus, ACS and ACO are the rate-limiting enzymes involved in this biosynthetic pathway. To reduce the ethylene accumulation through regulating the expression of genes encoding ACS and ACO enzymes might contribute to delay fruits and vegetables senescence (21, 114). Exogenous application of ABA induced flavanols and anthocyanin accumulation to promote the fruit coloration in fruits, including apple, grape, tomato, and litchi (115–118). Meanwhile, JA and SA have been suggested to be involved in the disease resistance during postharvest period (15, 16, 119, 120). MeJA induced the expression of JA synthesis genes, increased the allene oxide cyclase (AOC) activity, and thereby resulting in high endogenous JA generation (119). Nevertheless, DIECA treatment reduced the endogenous levels of JA, and AOC and 12-oxo-phytodienoic acid reductase activities. Then, a significant correlation between JA and chlorophyll content was observed in broccoli flowers, and that was the important reason for broccoli postharvest yellowing (119). Besides, SA-mediated defense response was involved in litchi downy blight possibly *via* modulating fruit senescence (120). Other hormones, such as auxins, cytokinins (CK) or GAs, are usually at very low contents and attributed to the anti-senescence properties as well (74, 121, 122).

**Figure 1:**
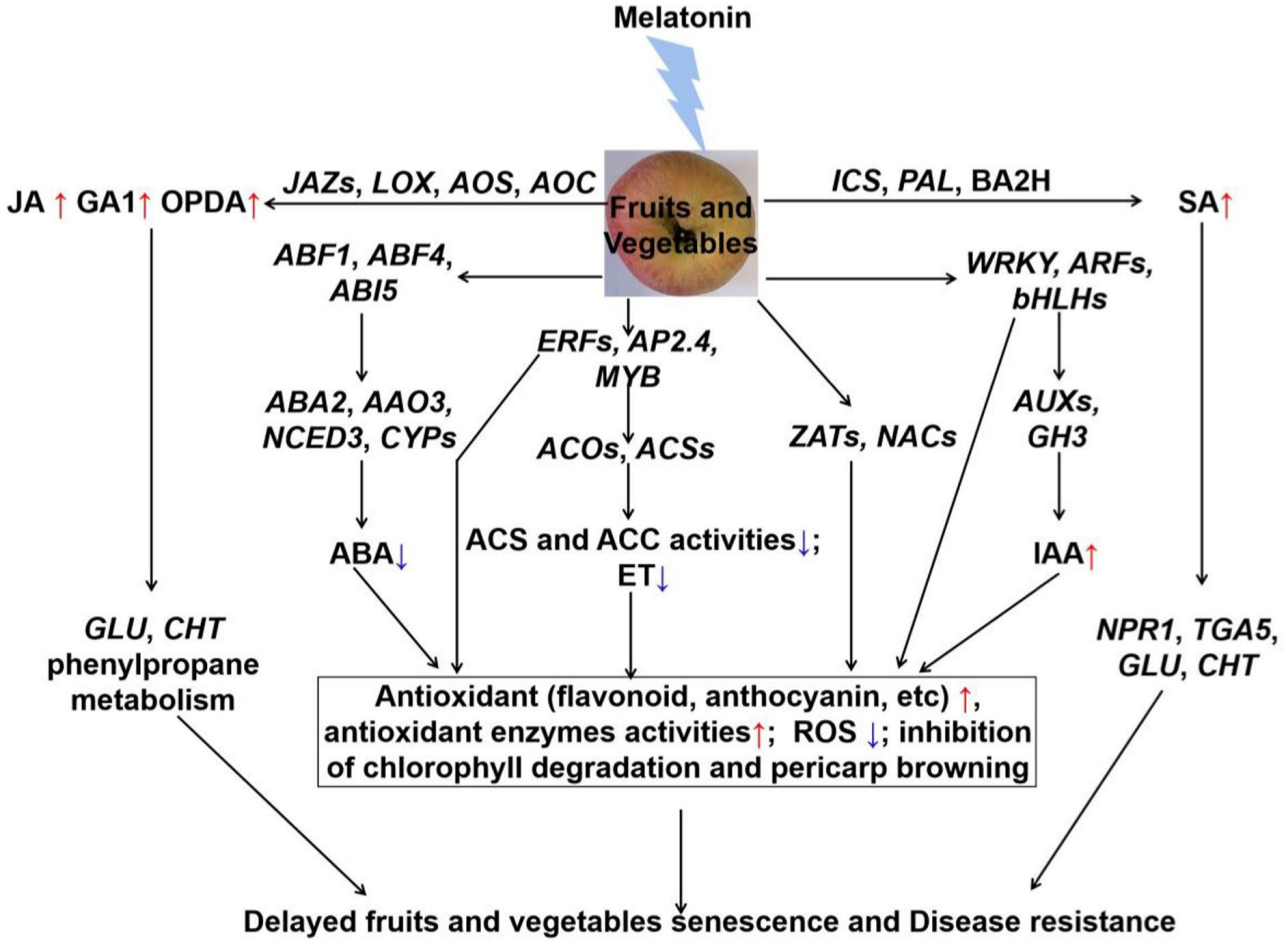
The crosstalk between melatonin and hormones of fruits and vegetables during the postharvest period. Exogenous application of melatonin reduced the ABA and ET content, and increased IAA content through regulating the related transcription factors and synthetic genes. Then, the significant antioxidants and antioxidant enzymes activities were induced to reduce the ROS accumulation, and chlorophyll degradation and pericarp browning were also inhibited. Besides, exogenous application of melatonin induced the JA and SA signaling pathways, and activated the proteins in the defense system to enhance the disease resistance in fruits and vegetables during storage. Jasmonic acid (JA), gibberellin 1 (GA1), 12-oxo-phytodienoic acid (OPDA), jasmonate ZIM-domain (JAZ), phenylalanine ammonia lyase (PAL), benzoic acid 2-hydroxylase (BA2H), lipoxygenase (LOX), allene oxide synthase (AOS), allene oxide cyclase (AOC), isochorismate synthase (ICS), salicylate (SA), non-expresser of pathogenesis-related genes 1 (NPR1), basic/leucine zipper-type transcription factor (TAG5), β − 1,3-glucanase (GLU), chitinase (CHT), abscisic acid (ABA), ABRE-binding factor (ABF), ABA-insensitive (ABI); 9-cis-epoxycarotenoid dioxygenase (NCED), aldehyde oxidase (AAO), ethylene-response factor (ERF), adipocyte protein (AP), v-myb avian myeloblastosis viral oncogene homolog (MYB), zinc finger protein (ZAT), NAM/ATAF/CUC (NAC), basic/helix–loop–helix (bHLH), 1-aminocyclopropane-1-carboxylic acid (ACC), ACC oxidase (ACO), ACC synthase (ACS), ethylene (ET), auxin response factor (ARF), indole-3-acetic acid-amido synthetase (GH3), indole-3-acetic acid (IAA).

Many studies have confirmed the role of melatonin in modulating hormone levels during fruits and vegetables postharvest period (Figure 1) and (123). Melatonin can significantly delay fruit and vegetables senescence through inhibiting ET and ABA accumulation. For example, exogenous application of melatonin inhibited the expression of *ACSs* and *ACOs* genes, and reduced ethylene production to delay the banana and tomato fruits color through (124, 125). It significantly down-regulated the expression of ET synthetase genes (*PcACS* and *PcACO*), reduced ethylene production and rates of respiration, then thereby delaying senescence in pear fruit (126). Correspondingly, melatonin also down-regulated the expression of ET transcription factors (*AdERF4*, *AdERF74*, and *AdERF75*), and inhibited the ET release in kiwifruit during the storage period (127). Interestingly, research studies have showed that exogenous application of melatonin repressed the expression of *BrABF1*, *BrABF4*, *BrABI5* (128). They binded to the promoters of ABA biosynthetic genes (*BrNCED*, *BrABA2*, and *BrAAO*) and chlorophyll catabolic genes, and regulated the expression levels of above genes, thus resulting in a low endogenous ABA level (128). Therefore, melatonin regulated the inhibition of Chinese flowering cabbage senescence by the suppression of *ABFs*-modulated ABA synthesis and chlorophyll degradation (128). Furthermore, exogenous application of melatonin reduced both ET and ABA contents to modulate the softening through inhibiting the activities of ACS, ACO, and 9-cis-epoxycarotenoid dioxygenase (NCED) in “Guifei” mango fruit (73). Additionally, exogenous application of melatonin induced the expression of JA synthesis genes (*VaLOX*, *VaAOS*, and *VaAOC*), and promoted JA accumulation (129). Hence, melatonin modulated the jasmonic acid signaling pathway to enhance the postharvest disease resistance of blueberries fruit (129). Similarly, generation of SA was also promoted by exogenous application of melatonin in tomato. Afterwards, the increase of activities of chitinase (CHI) and β-1,3-glucanase (GLU) inhibited tomato gray mold development, which caused by *B. cinerea* (130). Besides, after melatonin treatment for 4 days, GA1 had a sharp increase, and no differences were observed in the content of GA3, GA4, and GA7 in *Angeleno* plums during postharvest decay (70). Furthermore, it was also suggested that *WRKY*, *MYB*, *ERF*, *ARF* and *bHLH3* transcription factors were mainly involved in auxin and ethylene signalings in postharvest banana fruit peel (131, 132). These transcription factors were also beneficial to maintain redox homeostasis (133). Some others, such as auxin and mitogen-activated protein kinase (MAPK) signaling pathway, might be involved in melatonin-regulated fruits and vegetables postharvest preservation and/or disease resistance during the storage period. In summary, an appropriate amount of melatonin can prolong fruits and vegetables senescence shelf life by regulating the release of ET, ABA, SA, and etc. Additionally, more genetic evidence needs to be explored in future study.

## The crosstalk between melatonin and signal molecules (NO, H_2_S, and ROS) in the postharvest protection of fruits and vegetables during storage period

Numerous studies showed that signal molecules, such as ROS, NO, and H_2_S, play key roles in resistances to biotic and abiotic damages in plants (134–139). Recent studies have shown that there are interactions between melatonin (applied exogenously) and the signalig molecules (37, 40–42, 126, 130, 140). For example, our previous studies revealed that H_2_O_2_ signaling was required for melatonin-promoted root growth and melatonin-improved salinity tolerance in alfalfa and Arabidopsis, respectively (37, 38). NO signaling was also involved in melatonin-regulated salinity tolerance in *Brassica napus* L. and sunflower seedlings (140, 141). Furtherly, melatonin induced H_2_S generation through increasing L-/D-cysteine desulfhydrase (LCD/DCD) activity. Similarly, it also stimulated NO generation. However, the H_2_S and NO induced by melatonin were inhibited by H_2_S scavenger (hypotaurine, HT) and NO scavenger (2-(4-carboxyphenyl)-4,4,5,5-tetramethylimidazoline-1-oxyl-3-oxide, cPTIO), respectively. Therefore, the H_2_S and NO jointly were participated in the melatonin-enhanced salinity tolerance in cucumber (34). In fact, the complex regulatory function of melatonin and its crosstalk with H_2_O_2_, NO and H_2_S is existed in many cases.

**Figure 2:**
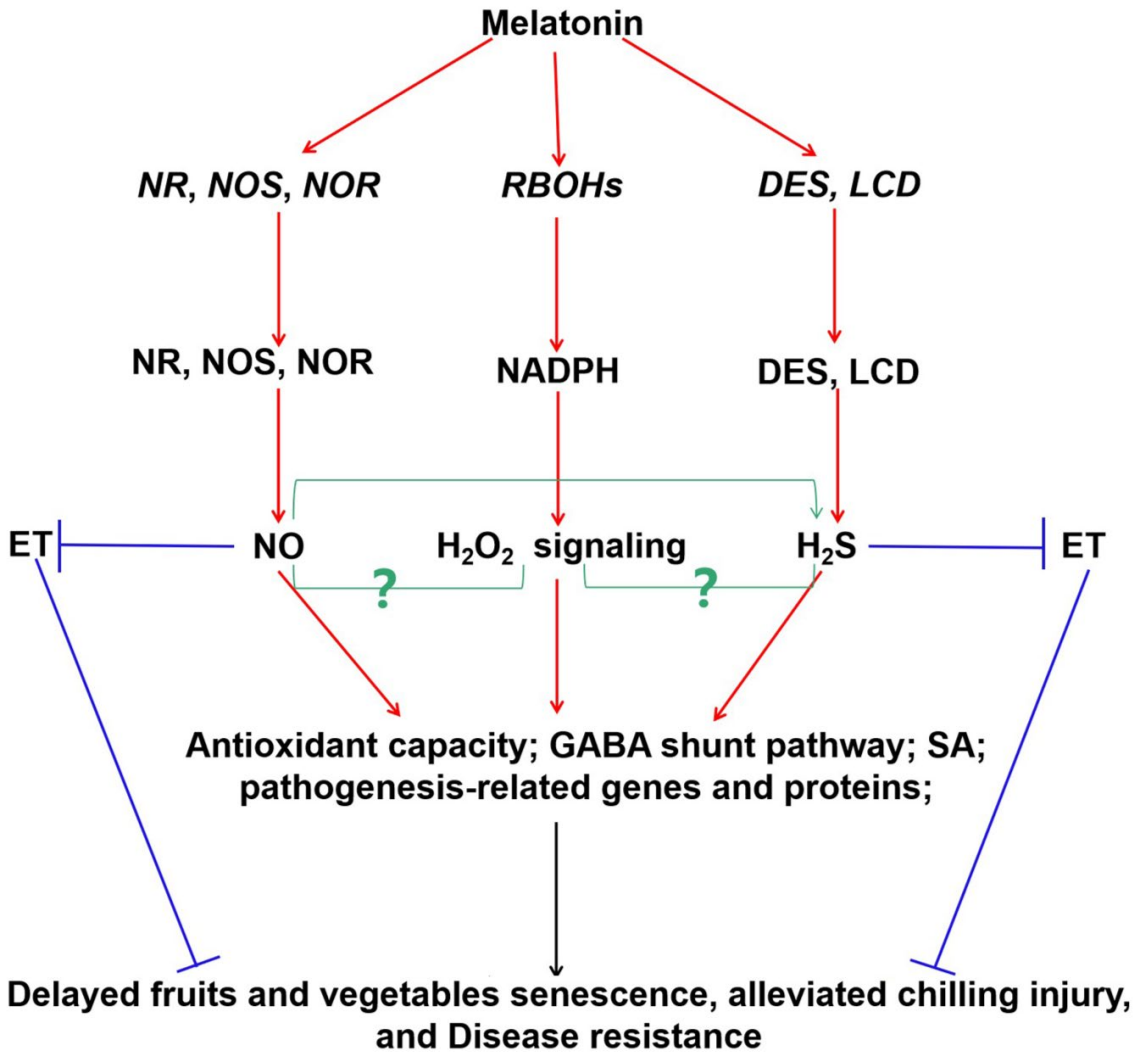
Probable integrative model of melatonin and signaling molecules (H_2_O_2_, NO, and H_2_S) in postharvest protection of fruits and vegetables during storage period. Increasing evidences showed that melatonin enhanced the expression of *RBOHs*, *NR*, *NOS*, *NOR*, *DES*, and *LCD* genes, and induced H_2_O_2_, NO, and H_2_S generation, thereby activating the signaling pathways in fruits and vegetables. Besides, hormones were also involved in these pathways to regulate fruits and vegetables quality. Interaction between NO and H_2_S was also suggested. The relationships between the H_2_O_2_ signaling and NO/H_2_S in postharvest protection of fruits and vegetables are still largely unknown (green arrow, yet largely unknown). Red arrow, induced; blue arrow, inhibited. Respiratory burst oxidase homologs (RBOHs), nitrate reductase (NR), nitric oxide synthase (NOS), desulfhydrase, (DES), *L*-cysteine desulfhydrase (LCD), nitric oxide (NO;), hydrogen peroxide (H_2_O_2_), hydrogen sulfide (H_2_S), ethylene (ET), salicylic acid (SA), γ-aminobutyric acid (GABA).

Interestingly, these signal molecules were also involved in exogenous melatonin-modulated fruits and vegetables postharvest protection, and thus improving their quality and yield (Figure 2) and (72, 142, 143). For instance, exogenous melatonin treatment rapidly elicited ROS burst. These ROS acted as signaling molecules to enhance SA accumulation and improve the expression of related defense genes in cherry tomato fruit during the storage (94). In litchi fruit, exogenous application of melatonin activated the NR and NOS activities and triggered NO accumulation (142). Endogenous NO mediated the melatonin-enhanced cold tolerance *via* regulation of redox status (142). Similarly, exogenous melatonin increased NOS activity, and induced endogenous NO production to maintain normal mitochondrial function in lotus seeds (144). Besides, it also induced *NOS* gene expression and enzyme activity to keep safe membrane integrity in tomato fruit (145). Furthermore, H_2_S has been reported to regulate the process by delaying senescence (146). However, more studies should be investigated on the crosstalks among melatonin, NO, and H_2_S in the postharvest preservation of fruits and vegetables using pharmacological, genetic, and proteomic approaches.

## Crosstalk between the RBOH-regulated ROS signaling and melatonin in the postharvest protection of fruits and vegetables during storage period

Previous studies suggested that melatonin is a potent free radical scavenger, and reacts with ROS *via* the addition of a hydroxyl group (-OH) in position 2, 4, or 6 to form a family of molecules (147). Among the hydroxymelatonin metabolites, 2-hydroxymelatonin (2-OHMel) and 4-hydroxymelatonin (4-OHMel) were found in 24 plant species and predicted to have the antioxidant protection (147–149). For example, 4-OHMel reacted with ROO• about 200 times faster than trolox. Furthermore, ROS act as key signaling molecules at low concentrations in regulating plant biotic and abiotic stress (150, 151). Recent studies have shed new light on the interactions of melatonin and ROS in higher plants development and growth (37, 38, 41). For example, Bian et al. (111) identified that melatonin acted as upstream signaling of ROS to facilitate lateral root development. Besides, the phytomelatonin receptor (PMTR) sensed and binded with melatonin to release G-protein α (Gα), and activated Ca^2+^ signaling. Afterwards, the Ca^2+^ signaling activated H_2_O_2_ production, while H_2_O_2_ worked with Ca^2+^ signaling to induce the expression of cell cycle regulatory genes, and thereby promoting the lateral root development.

Previous reviews summarized the pathways of ROS generation in plant organs, including cell membrane, peroxisome, mitochondria, chloroplast, apoplast, and etc (150, 151). Among these, respiratory burst oxidase homolog (RBOH) proteins localize on plasma membrane, and encode the NADPH oxidases, which associate with the signal transduction (152). There are several *RBOHs* genes encoding NADPH oxidase in various plants (150, 151). Recently, many studies have revealed the vital roles of *RBOH*-regulated ROS signaling in melatonin-enhanced plant abiotic stress tolerance (41). Furthermore, it is necessary to balance intracellular ROS homeostasis to maintain to the quality of postharvest fruits and vegetables. Recently, the functions of H_2_O_2_ signaling in melatonin-mediated fruits and vegetables postharvest protection were also preliminarily studied (Figure 2) and (72, 130, 132, 153, 154). For example, O_2_^·−^ and H_2_O_2_ generation of cherry tomato fruit increased to a maximum by exogenous melatonin treatment at 12 h and 36 h, respectively, and then decreased during the storage period (130). Exogenous melatonin treatment significantly up-regulated the expression of respiratory burst oxidase homolog protein B (*RbohB*) gene, which accelerated the response signaling in banana peel in banana during postharvest storage period (132). Similarly, melatonin treatment also up-regulated the *RBOH1* expression in tomato, however, it was significantly attenuated by treatments of diphenyleneiodonium (DPI, an NADPH oxidase inhibitor) and dimethylthiourea (DMTU, a ROS scavenger) (153). Exogenous melatonin elevated O_2_^·−^ and H_2_O_2_ accumulation by upregulating the *SlNOX* expression and NOX activity for the first 36 h in cherry tomato fruit during storage (94). These results were further confirmed by the transcriptome analysis in cherry tomato fruit (155). Besides, the positive crosstalks between melatonin and H_2_O_2_ have also been observed in apple and strawberry fruits against *Diplocarpon mali* infection and decay, respectively (51, 156). Moreover, SA signaling acted as the downstream pathway of the crosstalk between melatonin and H_2_O_2_ signaling to modulate the postharvest protection of fruits and vegetables during storage period (94, 156). Therefore, ROS generation-induced transiently by melatonin serve as the key signal in fruits and vegetables, especially in resistance to various diseases. However, it is important to further clarify the roles of this crosstalk on the quality and extending storage times in diverse fruits and vegetables species.

## Conclusion and perspectives

Melatonin is ubiquitous in fruits and vegetables. This reviews describes the changes of melatonin content and synthesis sites in fruits and vegetables during the postharvest period. Exogenous melatonin can increase endogenous melatonin accumulation, alleviate the weight loss, fruit firmness decrease and discoloration, reduce the decay incidence, decay and disease index, and improve the quality of fruits and vegetables. In addition, it increases GSH, AsA, DHA, anthocyanins, carotenoids, and total flavonoid and phenols contents, and decreases MDA, H_2_O_2_, and O_2_^•–^ contents. It has also been noted that melatonin enhances the CAT, SOD, APX, GR, GPX, DHAR, and MDHAR activities to improve the antioxidant capacity. Application of exogenous melatonin increases proline content and decreases the membrane lipid peroxidation to protect cell membrane integrity in fruits and vegetables during the cold storage. Furtherly, exogenous melatonin regulates hormones, such as ethylene, salicylic acid, and abscisic acid, to delay postharvest senescence and protect fruits and vegetables aganist bacterial invasion. However, the effective concentrations of melatonin are different for postharvest protection of different fruits and vegetables species. Therefore, it is important to use the appropriate melatonin concentrations to prolong fruits and vegetables postharvest shelf life.

ROS signaling during fruit and vegetable ripening has been extensively studied (147). Recently, several studies revealled that ROS signaling is involved in melatonin-modulated fruits and vegetables post-harvest preservation. In particular, the vital role of RBOHs-regulated H_2_O_2_ generation during these processes are shown. However, there are still many questions that should be characterized to understand the crosstalk of melatonin and ROS. For example, it is necessary to focus more attention on the signaling role of ROS produced by PAO in melatonin-modulated fruits and vegetables post-harvest preservation in future studies. Since the transmembrane receptor of melatonin (PMTR1/CAND2) were found in plants, researches focus on the mechanisms that the interaction between *PMTR1/CAND2* and Gα subunits acts on the expression of the *RBOHs* in plant responses to abiotic stress (56, 71, 157). In this review, it is urgent to deeply study whether or how Gα directly regulates the crosstalk between melatonin and reactive oxygen species in fruits and vegetables post-harvest preservation.

## Author contributions

NL, KZ, QY, and QG: writing—original draft preparation. XZ: writing—provided deeply discussion and sorted out the references. MM and ZC: writing—review and editing. All authors have read and agreed to the published version of the manuscript.

## Funding

This research was mainly supported by the Natural Science Foundation of Anhui Province (No. 2008085MC101), Anhui Province Innovation Team of Authentic Medicinal Materials Development and High Value Utilization (No. 2022AH010080), Talent Research Fund Project of Hefei University (No. 18-19RC13), Anhui Provincial First-class Professional Construction (Biotechnology and Food Quality and Safety), and the Natural Science Foundation of Anhui Educational Committee (No. KJ2017ZD36).

## Conflict of interest

The authors declare that the research was conducted in the absence of any commercial or financial relationships that could be construed as a potential conflict of interest.

## Publisher’s note

All claims expressed in this article are solely those of the authors and do not necessarily represent those of their affiliated organizations, or those of the publisher, the editors and the reviewers. Any product that may be evaluated in this article, or claim that may be made by its manufacturer, is not guaranteed or endorsed by the publisher.
